# Glycine Receptors in Spinal Nociceptive Control—An Update

**DOI:** 10.3390/biom11060846

**Published:** 2021-06-06

**Authors:** Hanns Ulrich Zeilhofer, Karolina Werynska, Jacinthe Gingras, Gonzalo E. Yévenes

**Affiliations:** 1 Institute of Pharmacology and Toxicology, University of Zurich, Winterthurerstrasse 190, CH-8057 Zürich, Switzerland; karolina.werynska@pharma.uzh.ch; 2 Institute of Pharmaceutical Sciences, Swiss Federal Institute of Technology (ETH) Zurich, Vladimir Prelog Weg, CH-8093 Zürich, Switzerland; 3 Drug Discovery Network Zurich, University of Zurich and ETH Zurich, Winterthurerstrasse 190, CH-8057 Zürich, Switzerland; 4 Homology Medicines Inc., 1 Patriots Park, Bedford, MA 01730, USA; jgingras@homologymedicines.com; 5 Department of Physiology, University of Concepción, Concepción 4070386, Chile; gyevenes@udec.cl; 6 Millennium Nucleus for the Study of Pain (MiNuSPain), Santiago 8320000, Chile

**Keywords:** glycine, GABA, pain, inhibition, spinal cord, dorsal horn, hyperalgesia, allodynia, circuit, mouse

## Abstract

Diminished inhibitory control of spinal nociception is one of the major culprits of chronic pain states. Restoring proper synaptic inhibition is a well-established rational therapeutic approach explored by several pharmaceutical companies. A particular challenge arises from the need for site-specific intervention to avoid deleterious side effects such as sedation, addiction, or impaired motor control, which would arise from wide-range facilitation of inhibition. Specific targeting of glycinergic inhibition, which dominates in the spinal cord and parts of the hindbrain, may help reduce these side effects. Selective targeting of the α3 subtype of glycine receptors (GlyRs), which is highly enriched in the superficial layers of the spinal dorsal horn, a key site of nociceptive processing, may help to further narrow down pharmacological intervention on the nociceptive system and increase tolerability. This review provides an update on the physiological properties and functions of α3 subtype GlyRs and on the present state of related drug discovery programs.

## 1. Introduction

**Figure 1:**
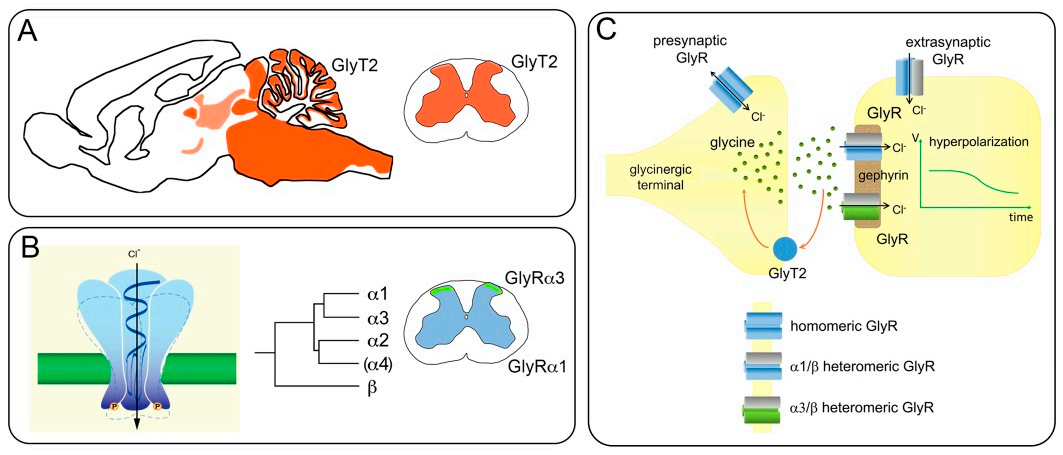
Principles of glycinergic inhibition. (**A**) Schematic sagittal mouse brain section illustrating the distribution of glycinergic innervation detected by GlyT2 staining (in red). High density innervation is found in the brainstem with particularly dense expression in the medulla oblongata and pons, and generally weaker expression in the midbrain and parts of the thalamus. Dense innervation is also found in the cerebellar cortex, the inferior colliculus, and the mesencephalic trigeminal nucleus. Cortex and hippocampus are virtually devoid of glycinergic innervation. In the spinal cord, glycinergic innervation is very widespread, with slightly less expression observed in the most dorsal laminae I and II. (**B**) Glycine receptors are chloride permeable heteropentameric ion channels, composed from a repertoire of five (in humans four) subunits each encoded by a separate gene (α4 is a pseudogene in humans). Each subunit is composed of a large extracellular domain followed by 4 transmembrane segments connected by loop structures and a short extracellular C-terminus (see also Figures 2A and 4). In the spinal cord, α1 subunit immunoreactivity (blue) is found throughout the grey matter, but α3 subunit expression (green) is highly and specifically enriched in lamina II. (**C**) Schematic representation of a glycinergic synapse. Glycine is released from a glycinergic terminal and binds to postsynaptic α/β heteromeric receptors anchored to the postsynaptic scaffolding protein gephyrin. Their activation by synaptic release causes an inhibitory postsynaptic potential. Glycine receptors are also found at extrasynaptic and presynaptic sites. Presynaptic glycine receptors lack β subunits and are not clustered by gephyrin. Activation of extrasynaptic glycine receptors cause a tonic inhibitory membrane current, whereas the activation of presynaptic glycine receptors may enhance transmitter release.

Inhibitory neurotransmission plays a crucial role in the maintenance of a physiologically meaningful state of pain sensitivity and helps separating innocuous and noxious signal relay. Following injury and inflammation, reduced inhibition contributes to the phenomena of hyperalgesia (an increased sensitivity to input from nociceptive fibers) and allodynia, which describes a painful sensation elicited by input from non-nociceptive fibers. While both phenomena may help protect injured tissue from further damage and may foster its healing, under unfortunate conditions they outlast the healing process and may then severely compromise the quality of life of affected patients. The spinal dorsal horn is a key site for endogenous pain control and maladaptive plasticity which underlies many chronic pain conditions. Various processes triggered by peripheral inflammation or nerve damage compromise synaptic inhibition at this site and in corresponding brainstem areas. Among these processes are alterations in the excitatory drive to inhibitory dorsal horn neurons [1], a compromised electrochemical gradient of chloride ions [2,3], and altered responsiveness of inhibitory neurotransmitter receptors [4]. While the first two mechanisms are triggered by peripheral nerve damage and affect both GABAergic and glycinergic inhibition, peripheral inflammation has a specific impact on the function of dorsal horn GlyRs. General aspects of glycinergic neurotransmission are illustrated in Figure 1.

## 2. GlyRs in Inflammatory Hyperalgesia and Allodynia

Cyclooxygenase-2 (COX-2) derived prostaglandin E2 (PGE_2_) is a pivotal mediator of inflammation and inflammatory hyperalgesia. In response to peripheral inflammatory insults, PGE_2_ is not only produced in the periphery at the site of inflammation, but also in the CNS, especially in the spinal dorsal horn, where it contributes to the phenomenon of central sensitization. A series of reports published between 2002 and 2005 established the critical role of α3 GlyRs in inflammation and PGE_2_-mediated central sensitization. PGE_2_, but not other prostaglandins including PGD_2_, PGI_2_, or PGF_2α_, reduces glycinergic synaptic transmission in superficial dorsal horn neurons through a postsynaptic mechanism involving the activation of EP2 receptors, production of cAMP, and subsequent activation of protein kinase A (PKA) [5]. This inhibitory effect was lost in mice that lack a specific GlyR subtype defined by the inclusion of the α3 subunit in the pentameric receptor complex (GlyR α3) [6]. The expression of this subunit in the spinal cord is largely confined to layer II of the dorsal horn, which is also known as the *substantia gelatinosa*. This site constitutes the termination area of most nociceptive fibers arriving in the CNS from the periphery. A systematic in silico screen of the GlyR α3 protein sequence revealed a strong consensus site for PKA-dependent phosphorylation in the large intracellular loop between transmembrane segments 3 and 4. This consensus site comprises the amino acid sequence arginine-glutamate-serine-arginine (RESR), in which the serine at position 346 constitutes the actual site of phosphorylation. The critical role of this site for PGE_2_ triggered GlyR α3 phosphorylation was supported by results obtained in HEK293 cells. Heterologous expression of EP2 and α3 GlyRs reconstituted the inhibition of glycinergic membrane currents by PGE_2_. Replacing the serine residue at position 346 by an alanine, an amino acid of similar size but lacking the OH group required for phosphorylation, prevented inhibition by PGE_2_ [6].

The availability of mice lacking α3 GlyRs allowed assessing the contribution of α3 GlyRs and their regulation by PKA to baseline nociception and different forms of hyperalgesia. GlyRα3 deficient mice behaved normally in tests of baseline nociception (noxious heat or punctate mechanical stimulation with von Frey filaments). This lack of a pronociceptive phenotype may be linked to unaltered baseline glycinergic neurotransmission in GlyR α3 deficient mice and may hint at a compensatory up-regulation of other GlyR subunits. Pronounced alterations in the development of hyperalgesia became apparent when the mice were challenged with peripheral inflammation. In wild-type mice, subcutaneous injection of complete Freund’s adjuvant or with the yeast extract zymosan A induces thermal and mechanical hyperalgesia, which lasted for several days to weeks depending on the amount injected. GlyR α3 deficient mice showed strongly reduced thermal and mechanical hyperalgesia especially during the later phases of inflammation. A virtually identical phenotype was observed in a side-by-side comparison of GlyR α3 deficient mice and mice lacking prostaglandin EP2 receptors [7]. 

**Figure 2:**
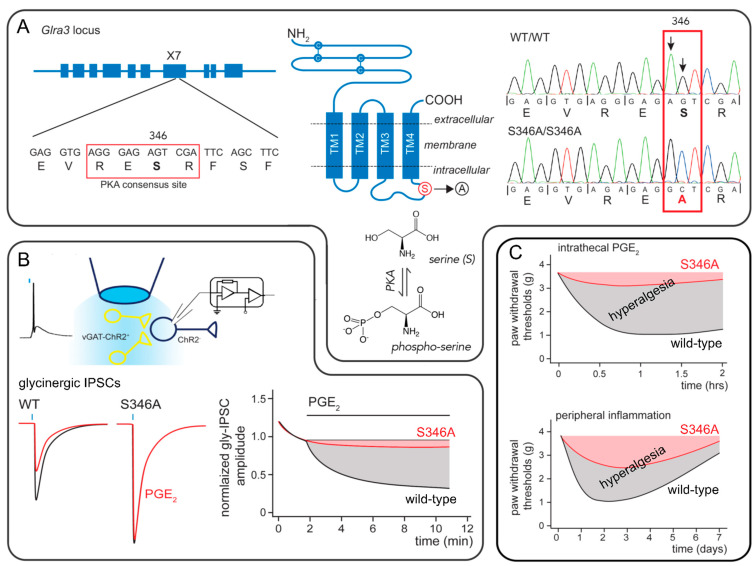
PKA-dependent phosphorylation on α3 GlyRs. (**A**) Left: Exon 7 (X7) of the *Glra3* gene contains a DNA sequence encoding for a strong consensus site for PKA-dependent phosphorylation. Middle: This site consisting of the four amino acids RESR (positions 344-347) is located in the long intracellular loop of the α3 subunit. Mutation of serine at position 346 (S346) to an alanine prevents phosphorylation at this site. Left: CRISPR-Cas technology was used to introduce this mutation (S346A) into the genome of mice. Pherograms showing the DNA sequencing results of the part of X7 that contains the PKA consensus site in wild-type mice and homozygous S346A point mutated mice. (**B**) Top: Patch-clamp recordings were made for excitatory neurons in spinal cord slices obtained from wild-type and homozygous S346A point mutated mice, which both carried a channelrhodopsin-2 transgene expressed in inhibitory neurons for optogenetic activation. Brief pulses of blue light evoke action potentials in inhibitory neurons and as a consequence inhibitory postsynaptic currents (IPSCs) in synaptically connected neurons. Bottom left: Glycinergic IPSCs were inhibited by PGE_2_ in wild-type but not S346A mice. Right: Time course of the amplitudes of normalized glycinergic IPSCs before and during superfusion of the slices with PGE_2_. (**C**) Top: Hyperalgesia evoked by intrathecal injection of PGE_2_ was completely absent in S346A point mutated mice. Bottom: Hyperalgesia triggered by subcutaneous injection of the yeast extract zymosan A was strongly reduced in the point-mutated mice.

Although the reconstitution experiments in HEK293 cells supported the critical role of a direct PKA-dependent phosphorylation of α3 GlyRs, unequivocal proof for its relevance to in vivo hyperalgesia was still lacking. The recent generation of a genetically engineered S346A point mutated mouse line allowed filling this gap (Figure 2). This mouse carries a serine to alanine amino acid exchange at position 346 (S346A mutation) in the PKA consensus sequence of the GlyR α3 subunit [8]. Electrophysiological recordings from *substantia gelatinosa* neurons in spinal cord slices of these mice demonstrated not only that the point-mutated α3GlyRs were resistant to inhibition by PGE_2_ but also confirmed the critical contribution of phosphorylation at this site to inflammatory hyperalgesia. Mice carrying the S346A point mutation were resistant to the hyperalgesic effects of intrathecally injected PGE_2_ and developed much less hyperalgesia after injection of zymosan A into one hindpaw.

Besides inflammation, neuropathy is another major source of chronic pain and hyperalgesia. A contribution of COX-2 or PGE_2_ to neuropathic pain has been proposed [9] but is still rather controversial. GlyR α3 deficient mice have previously been tested in the constriction injury model of neuropathic pain [10]. Both wild-type mice and GlyR α3 deficient mice developed prolonged thermal and mechanical hyperalgesia following the constriction injury of the sciatic nerve, suggesting that the phosphorylation of GlyR α3 subunits is dispensable for the development of hyperalgesia following peripheral nerve injury. This finding was recently confirmed in experiments with S346A point mutated mice, which also developed unaltered hyperalgesia following peripheral nerve injury [8]. It should be stressed that the normal development of neuropathic hyperalgesia in the GlyR α3 deficient or S346A point mutated mice does not exclude that α3 GlyRs still control neuropathic hyperalgesia; it only means that these receptors are not phosphorylated or inhibited in the course of neuropathy. In fact, two relatively recently developed GlyR modulators, AM-1488 [11] and 5-desoxy-THC/DH-CBD [12], reduce neuropathic pain in rodents (for a more detailed discussion of the action of these molecules see Section 5).

Additional insights have also been gained into the molecular mechanisms that link phosphorylation at S346 to decreased glycinergic currents [13]. In HEK293 cells cotransfected with cDNAs encoding for GlyRα3 and EP2 receptors, application PGE_2_ led to a decrease in glycinergic membrane currents with no change in their inactivation kinetics or plasma membrane expression. In single channel recording experiments, activation of PKA progressively reduced single current amplitudes to about 66%, which is close to the inhibition of glycinergic synaptic currents observed in mouse spinal cord slices [5,8,14]. Introducing the phospho-mimicking serine to glutamate (S→E) mutation reduced single channel conductance to a similar degree with no effect on single channel open probability. Although previous work had suggested that S346 phosphorylation elicits structural changes in the α3 glycine-binding site [15], the introduction of the S346E mutation had no effect on the potency (EC_50_) of GlyR currents. Substituting S346 with phospho-deficient alanine left the single channel amplitude and open probability almost unaffected. 

## 3. Circuit Aspects of Glycinergic Control of Spinal Nociception

**Figure 3:**
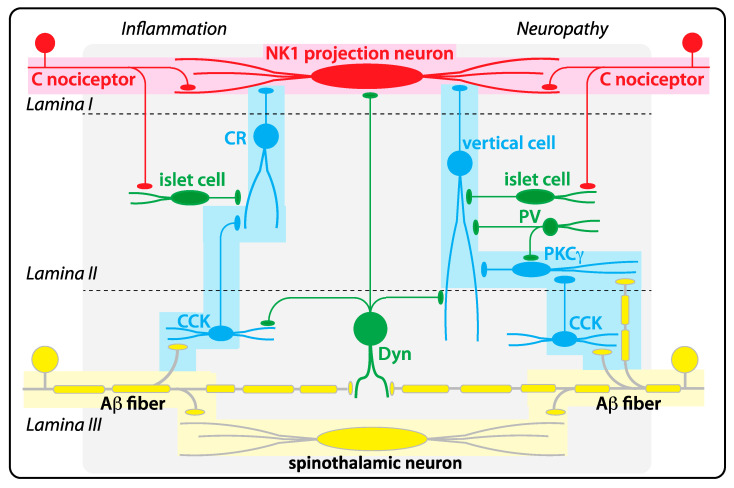
Simplified model of the dorsal horn neuronal circuits of allodynia recruited during inflammation and neuropathy. Under healthy conditions, relay pathways for noxious stimuli (red) and innocuous tactile stimuli (yellow) are strictly separated. Noxious stimuli enter the superficial layers of the dorsal horn via nociceptive C fibers, which activate neurokinin 1 receptor positive (NK1) projection neurons located in lamina I. Tactile input is conveyed by myelinated Aβ fibers, which activate spinothalamic projection neurons in the deep dorsal horn. Polysynaptic connections between both modalities are preexisting (blue) but normally silenced by glycinergic and GABAergic input. In inflammation (left), the pathway starts with cholecystokinin (CCK) positive excitatory interneurons that are activated by Aβ fibers and project to calretinin positive neurons (CR), which then excite NK1 projection neurons in lamina I. In neuropathy, the pathway also begins with cholecystokinin (CCK) positive interneurons, which activate PKCγ positive interneurons. The PKCγ interneurons the project to so-called vertical cells, which connect to lamina I NK1 projection neurons. PKCγ positive neurons can also be directly activated by Aβ fiber input. Several types of inhibitory interneurons (green) silence these polysynaptic pathways, among them are parvalbumin positive (PV) neurons, dynorphin positive interneurons (Dyn), and so-called islet cells. Dyn and PV neurons evoke postsynaptic responses with a strong glycinergic component. About half of the more superficially located islet cells release glycine in addition to GABA. Many of these inhibitory interneurons are activated by input from nociceptors or non-nociceptive Aβ fibers and thereby provide feed-forward inhibition. Schemes are based on [16,17].

To fully understand of the role of α3 GlyRs in spinal control of nociception, precise knowledge of the neural circuits controlled by these receptors is essential. On a very gross scale, spinal hyperalgesia may be viewed as an imbalance of excitatory nociceptive input and local inhibitory control. In reality, the situation is likely much more complex. Figure 3 depicts some of the polysynaptic pathways of the spinal dorsal horn which become functional in different pathological pain states.

The simplest mechanism by which GlyRs may contribute to spinal nociceptive control might be the direct inhibition of nociceptive output from the spinal cord. This output occurs mainly via projection neurons located in lamina I of the dorsal horn, which relay nociceptive signals to the brainstem. These neurons receive direct excitatory drive from nociceptors and are controlled by GABAergic and glycinergic input [18]. The glycinergic input to lamina I neurons is reduced in rats with inflamed paws [19]. Altered heat hyperalgesia in the α3 GlyR-deficient mice [6] may reflect changes in the glycinergic control of superficial dorsal horn neurons, as heat stimuli are primarily processed in this area.

However, compared to neurons of the deeper dorsal horn, projection neurons of lamina I receive relatively little spontaneous inhibitory input [18], potentially suggesting that GlyRs might primarily be relevant to sensory processing in the more complex circuits of the deeper dorsal horn. Glycinergic neuron somata are in fact more prevalent in the deeper dorsal horn layers (laminae III and deeper) than in lamina I and II [20]. Accordingly, postsynaptic glycine responses are also larger and more prevalent in the deep than in the superficial dorsal horn [21,22]. It should however be noted that even in lamina II, the glycinergic component of inhibitory postsynaptic currents still outweighs the GABAergic component [23]. This anatomical gradient likely bears functional implications, as the deep dorsal horn receives mainly tactile (Aβ fiber) input while the nociceptive (C and Aδ fiber) input dominates in the superficial dorsal horn. Impaired segregation of signal relay in the superficial versus deep dorsal horn is thought to underlie allodynia, the painful sensation evoked by tactile stimuli. It is believed to result from the abnormal activation of lamina I neurons by Aβ fiber input. Lamina projection neurons normally become activated only in response to noxious input. After blockade of GABA_A_ receptors and GlyRs, these neurons become excitable also by input from Aβ fibers through a polysynaptic pathway [24]. 

The first neuron type identified in this pathway were protein kinase Cγ (PKCγ) expressing excitatory interneurons [25], which are located at the border between lamina II and lamina III and hence at the interface of innocuous tactile and noxious input [26]. Subsequent work has identified several additional elements of this circuit (for a recent review on allodynia circuits of the dorsal horn, see also [16]). Very recent work suggests that several different pathways exist, which are differentially recruited in inflammatory of neuropathic pain states [17]. For several of these neuron types, the presence of GlyRs (as well as GABA_A_ receptors) on their surface has been directly demonstrated [25,27,28]. These GlyRs become activated in a feed-forward mechanism initiated by input from non-nociceptive tactile fibers [29]. It is believed that excitatory interneurons of these allodynia circuits are normally under strong inhibitory control, leading to the gating (closure) of the polysynaptic connection under normal conditions [30]. Consistent with this model, ablation of dorsal horn glycinergic neurons induces behavioral signs of allodynia and spontaneous discomfort in mice [23]. Similar phenotypes have also been observed after ablation or silencing of genetically defined subsets of glycinergic neurons, such as inhibitory parvalbumin and dynorphin neurons [31,32]. Despite these new insights, the specific location of α3 GlyRs on particular types of dorsal horn neurons and the subtypes of inhibitory interneurons that target α3 GlyRs are still unknown.

Inhibitory dorsal horn neurons do not only provide classical postsynaptic inhibition to intrinsic dorsal horn neurons, but also target axon terminals of primary sensory afferent nerve fibers. These terminals express GABA_A_ receptors but no GlyRs. Accordingly, glycine does not contribute to so-called primary afferent depolarization or presynaptic inhibition of primary afferent input. However, GlyRs reside on presynaptic terminals of central neurons, where they increase transmitter release. Such presynaptic GlyRs have first been found in neurons of the auditory brainstem, where their activation increases glycine release [33]. A similar action has later been reported for glycinergic input onto commissural neurons of the spinal dorsal horn [34]. The subunit composition of these presynaptic GlyRs is unknown. However, unlike postsynaptic GlyRs, presynaptic GlyRs may be homomeric receptors (i.e., lack β subunits) as presynaptic GlyRs are not clustered by gephyrin (see also Figure 1). Whether α3 subunits contribute to presynaptic GlyRs in the dorsal horn is unknown but experiments in hypoglossal motoneurons have shown that forskolin (cAMP)-induced facilitation of transmitter release was reduced in mice lacking α3 GlyRs [35].

## 4. Genetic Evidence of Glycinergic Pain Control in Humans

Direct evidence to support the presence of glycinergic pain control in humans is difficult to obtain given the present lack of compounds suitable for clinical testing in humans. A recent study in human patients suffering from inherited hyperekplexia provides however supporting evidence [36]. Most hyperekplexia patients carry homozygous (or compound heterozygous) loss-of-function mutations in GlyR genes (mainly α1 and β) or in the glycine transporter GlyT2, whose dysfunction leads to impaired loading of glycinergic terminals with glycine. The main symptom in these patients is an exaggerated startle response upon exposure to sudden sensory stimuli such as loud and abrupt noise or unexpected touch. Using a quantitative sensory testing battery, the recent study revealed in addition decreased pain thresholds in hyperekplexia patients.

While mutations in *GLRA1* and *GLRB* as well as in *SLC6A5* (encoding for GlyT2) are well-established causes of hyperekplexia, *GLRA3* has not been linked to any human disease yet. This may suggest that pharmacological targeting of α3 GlyRs may be relatively safe, but it also means that human genetic evidence supporting a specific role of α3 GlyRs is lacking. On a positive note, the PKA consensus site in the large intracellular loop of α3 GlyRs is conserved in humans. It is also noteworthy that the phenotype described in α3 GlyR deficient mice (reduced inflammatory pain) might not be easily detectable in humans. Furthermore, a screening of the human genetic variation database (www.ncbi.nlm.nih.gov/variation/) for loss of function (non-sense) variants revealed few hits. All of which occurred with frequencies too low to allow systematic clinical trials in such persons (for a discussion on this topic, see [8]).

## 5. Synthetic Glycine Receptor Modulators with Potential Analgesic Effects

**Figure 4:**
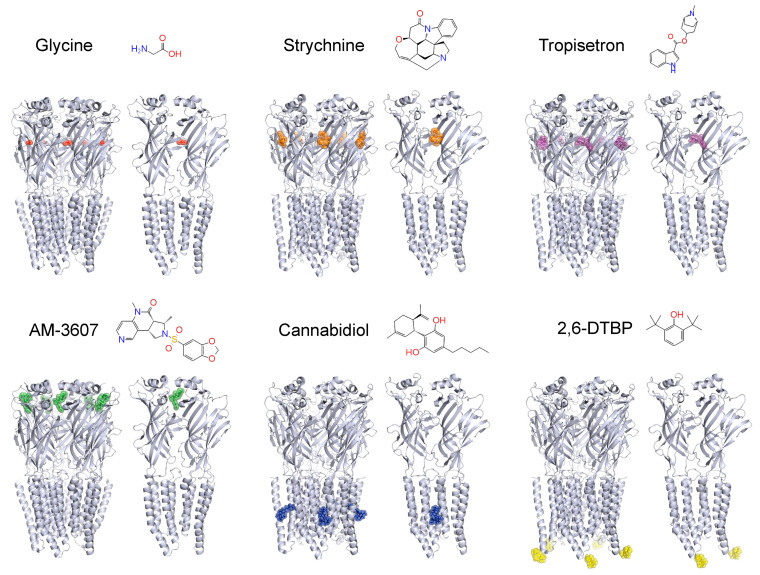
Modulators of α3 GlyRs. Structures of α3 GlyRs bound to the agonist glycine, the competitive antagonist strychnine, or the exogenous modulators tropisetron, AM-3607 (whose chemical structure is very similar to that of AM-1488), 2,6-di-tert-butylphenol (2,6-DTBP) and cannabidiol (CBD). To highlight the relevance of inter-subunit binding sites, models showing the interface of two adjacent α3 subunits are also shown. The interaction of tropisetron with the orthosteric site of α3 GlyRs was modeled according to [44]. The AM-3607 binding site was reconstructed as described in [11]. The models showing the CBD and 2,6-DTBP interaction with α3 GlyRs were generated based on [12,14]. The α3 GlyR structural coordinates (PDB ID: 5TIO and 5CFB) were taken from [11,45].

The rather selective expression of α3 GlyRs at a site critical for nociceptive processing and the phenotype of GlyR α3 deficient mice have sparked considerable interest in these receptors as targets for novel analgesics. Unlike the closely related GABA_A_ receptors, GlyRs have remained therapeutic orphans. Nevertheless, several drug companies (e.g., AMGEN [11,37,38] and Neusentis [39]) and academic groups [40,41,42] have recently reported the synthesis or identification of small molecule potentiators of GlyRs. In addition, AMGEN reported a series of pan (α1 and α3) and selective (α1 or α3) GlyR antibodies with functional (agonistic or antagonistic) activity profiles suggesting that selectivity within this receptor family can be achieved [43]. Figure 4 provides an overview over different small molecule GlyR activators and potentiators and their binding sites in the channel complex. 

Recent drug screening efforts at AMGEN have led to the discovery of novel glycine receptor potentiators [11,37,38]. Of particular interest, AM-1488, a highly selective positive allosteric modulator of α1 and α3 GlyRs acts through a high affinity binding (in the sub-micromolar range) to a newly discovered site in the large extracellular domain [11]. AM-1488 (20 mg/kg; p.o.) has been tested in a mouse spared nerve injury (SNI) model of neuropathic pain, where it was as effective as the reference compound pregabalin (30 mg/kg; p.o.), while its enantiomer was inactive at the same dose [11,37].

Other evidence supporting potential therapeutic benefit of GlyR potentiators comes from cannabinoid derivatives. Δ^9^-tetrahydrocannabinol (THC) modulates GlyR function, in addition to activating G protein coupled CB_1_ and CB_2_ receptors. Chemical modification of THC has led to the derivatives 5-desoxy-THC (identical to DH-CBD), 1-desoxy-THC, and di-desoxy-THC. Further, 5-desoxy-THC/DH-CBD and di-desoxy-THC are devoid of activity at CB_1_ and CB_2_ receptors but still bind to GlyRs. In addition, 5-desoxy-THC/DH-CBD acts as a positive allosteric modulator and di-desoxy-THC as its competitive antagonist of GlyRs [40]. In mouse inflammatory pain models, 5-desoxy-THC/DH-CBD exerted antihyperalgesic effects that were absent in α3 GlyR deficient mice but retained in CB_1_ and CB_2_ deficient mice [12]. 

Propofol (2,6-diisopropylphenol) is an intravenous anesthetic that targets primarily GABA_A_ receptors. At higher concentrations, it also acts as a GlyR modulator. The propofol derivative 2,6-di-tert-butylphenol (2,6-DTBP) is devoid of activity at the major GABA_A_ receptor subtypes [46] but retains activity at GlyRs [47] and possesses antihyperalgesic activity in neuropathic mice at high doses [14]. Critical for the interaction of propofol derivatives with GlyRs is a phenylalanine residue (F388 in the α3 subunit) in the large intracellular loop and close to the S346 phosphorylation site [48]. Interestingly, potentiation of synaptic GlyRs by 2,6-DTBP depends on the phosphorylation status of the GlyR α3 subunit. In mouse spinal cord slices, 2,6-DTBP prolonged the kinetics of glycinergic IPSCs only when α3 GlyRs were phosphorylated (“primed”) with PGE_2_ or when it was tested in spinal cord slices prepared from animals with inflamed paws [14]. 

Molecules with positive allosteric activity at GlyRs have also been isolated from a natural compound library generated from Australian and Antarctic marine invertebrates and algae [42,49,50]. These compounds have highly complex chemical structures hampering their artificial synthesis. Probably for this reason, in vivo activities have not been tested yet. Interestingly, one of the compounds specifically modulates α3 GlyRs with no activity at α1 GlyRs [49], suggesting that the development of α3 selective compounds is in principle feasible.

## 6. Other Compounds with Modulatory Actions at Glycine Receptors

GlyRs are in addition modulated by a number of endogenous molecules or synthetic compounds that either lack drug-like properties or activate primarily targets different GlyRs. Although such compounds are unlikely to be used therapeutically, they have led to the discovery of sites for allosteric modulation of different GlyRs. As such, they may provide starting points for drug discovery programs. Below, we provide a relatively short summary of this work. A more comprehensive coverage of this topic can be found elsewhere [51,52,53,54].

Endocannabinoids are lipid signaling molecules that primarily activate the G protein coupled cannabinoid CB_1_ and CB_2_ receptors. Arachidonoyl ethanolamide (AEA) and 2-arachidonoyl glycerol (2-AG) are pivotal endogenous activators of these receptors. Related arachidonoyl conjugates potentiate GlyR function with partially different effects on the different GlyR subtypes [55,56]. While neutral compounds such as AEA, N-arachidonoyl-serotonin, and N-arachidonoyl-dopamine potentiate α1, α2, and α3 GlyRs, the acidic compounds N-arachidonoyl-glycine, N-arachidonoyl-serine, N-arachidonoyl-L-alanine, arachidonic acid, and N-arachidonoyl-GABA potentiate α1 but inhibit α2 and α3 GlyRs [55]. Building on these differential effects has allowed the identification of relevant sites in the GlyR protein through the generation and analysis of chimeric GlyR constructs. These experiments revealed a relatively complex scenario with one relevant amino acid located in the extracellular domain (alanine at position 52 in α1), two amino acids in transmembrane segment 2 (glycine 254 in α1 and alanine 265 in α3), and in the large intracellular loop (lysine 385 in α1). Other work provides a detailed analysis of effects of the acyl chain length and numbers and sites of double bonds within the acyl chain on the modulation of GlyRs with different subunit combinations [57]. 

Tropeines are antiemetic drugs that act as antagonists at ionotropic serotonin (5-HT3) receptors. Two tropeines, MDL-72222 and tropisetron, also potentiate GlyR activity at nanomolar concentrations [58,59,60]. They bind to an interface between two α or one α and a β subunit in the extracellular domain. The effects of tropeines have been mainly explored on α1 GlyRs, and less in α3 GlyRs. A recent report explored the modulation of homopentameric α3 GlyRs by tropeines [44]. Tropisetron did not potentiate α3 GlyRs, but rather caused concentration-dependent inhibition in the low micromolar range. In silico docking confirmed that tropeines may bind to the extracellular domain of α3 GlyRs. Since tropisetron also displayed excusive inhibitory effects on α2 GlyRs [59], these data suggest a subunit-specific effect on α1 GlyRs. 

Zonisamide is an antiepileptic drug, which has recently been reported to facilitate activation of recombinant and native α1, α2, and α3 GlyRs at therapeutic concentrations [61]. Zonisamide has many potential therapeutic targets including voltage gated Na^+^ channels and T-type Ca^2+^ channels and it is unclear which target is responsible for its antiepileptic effect. Some evidence suggests that zonisamide possesses antihyperalgesic activity in different preclinical models [62]. This is, however, a common action also of anticonvulsant drugs including the gabapentinoids, carbamazepine, and phenytoin and it is hence unclear whether potential analgesia might originate from its modulatory activity at GlyRs. More remarkable is the absence of the potential side effects of GlyR modulation discussed above (muscle relaxation, respiratory dysfunction, addiction) [63]. 

Lastly, glycine receptor activity can also be modulated in a bidirectional fashion by zinc, where sub-micromolar concentrations (20 nM–1 µM) lead to potentiation and micromolar concentration (20–50 µM) to inhibition [64], reviewed in [65]). The biological presence of zinc documented in synaptic vesicles in the brain, suggest indeed that it bears unique neurotransmission modulation roles within the central nervous system [66,67,68,69]; reviewed in [65]). Thus, adding zinc chelators (such as compatible with the screening platforms selected) is highly recommended in future HTS-type screens to reduce the rate of false-positive hits moving-forward (see [70] for a comprehensive list of zinc chelators readily available).

## 7. Is Specificity for the α3 Subtype Preferred or Required?

This question cannot be answered yet [52]. GlyRs do not only control nociception but several other physiological functions. Best known among them is the control of motoneuron activity and hence muscle tone. In addition, GlyRs are abundant in the pre-Bötzinger complex, which controls respiration, in the ventral tegmental area (VTA) and the nucleus accumbens (NAc), which form the brain’s reward system, the retina and the auditory system. While pharmacological modulation of inhibitory transmission appears to have little impact on visual or auditory perception, effects on motoneuron activity, respiratory control, and addiction are areas of potential concern. Motoneurons virtually lack α3 GlyRs, suggesting that α3 selective modulators should be devoid of undesired muscle relaxation. In contrast, the pre-Bötzinger complex, the VTA, and the NAc contain α1 and α3 GlyRs [71,72,73,74,75,76]. Selectivity for the α3 subtype may protect from some of these potential undesired drug effects but it may also reduce efficacy as most GlyRs even in the substantia gelatinosa contain both α1 and α3 subunits [77]. While several lines of evidence strongly support that GlyR potentiators will reduce pathological hyperalgesia, it is currently uncertain whether subtype specific modulators should be preferred in drug discovery programs. With the recent progress in identifying selective GlyRs small and large molecules, it is only a question of time before highly selective and potent drug candidates can allow the field to test whether selectivity is preferred or required.
